# Supplementary material for: Exploring Transfer Potentials of the IMPROVEjob Intervention for Strengthening Workplace Health Management in Micro-, Small-, and Medium-Sized Enterprises in Germany: A Qualitative Study
Source: Int J Environ Res Public Health. 2023 Feb 24;20(5):4067. doi: 10.3390/ijerph20054067 (PMC10002393; doi:10.3390/ijerph20054067)
Supplement: Supplementary file 1 [file ijerph-20-04067-s001.zip › ijerph-2111431-supplementary.pdf]

International Journal of Environmental Research and Public Health

Article

Exploring Transfer Potentials of the IMPROVE*job* Intervention for Strengthening Workplace Health Management in Micro-, Small-, and Medium-Sized Enterprises in Germany: A Qualitative Study

Anke Wagner, Brigitte Werners, Claudia Pieper, Anna-Lisa Eilerts, Tanja Seifried-Dübon, Matthias Grot, Florian Junne, Birgitta M. Weltermann, Monika A. Rieger, and Esther Rind

Supplementary Material

Content of Supplementary Material

- **Table S1:** Interview guide for single interviews
- **Table S2:** Template for the documentation of the single interviews
- **Table S3:** Template for the documentation of the focus group discussion
- **Table S4:** Schedule of the focus group discussion
- **Table S5:** Items of the online questionnaire

Table S1: Interview guide for single interviews

Note: Translated to English by the authors for the purpose of publication. Original version was in German only.

Structure of the interviews	Content of the interviews
**1. Introduction**	• Welcome and thanks for participation<br>• Information about the IMPROVE*job* study<br>• Information about the interview (aim of the interview, presumed duration, request to freely describe your impressions, no right and wrong answers)<br>• Brief repeated information on data protection
**2. Short questions about the expert and the enterprise**	• Sector of your company?<br>• Number of employees in your company?<br>• How long have you been managing or working in the business/company?<br>• Does the business/company have other branches?<br>• Do you share the management of the business/company with a partner?<br>• Do you have any executive employees?<br>• Is your business/enterprise a family business?
3. **Interview questions in relation to job satisfaction**	Key question<br>• What factors and aspects do you think have an impact on job satisfaction?<br>Follow-up questions<br>• What could increase/improve your job satisfaction / that of your employees?<br>• When are you satisfied in the workplace?
4. **Interview questions in relation to team communication**	Key question<br>• What do you think are the requirements for good teamwork?<br>Follow-up questions<br>• What is your common understanding of teamwork?<br>• What enables your team to work well together?
**5. Interview questions in relation to leadership**	Key question<br>• What do you think is the importance of you as a "manager" for the satisfaction and health of your employees?<br><br>Follow-up questions<br>• How should managers perform their role in the best possible way?<br>• What prevents you from doing so?

**Structure of the interviews**	**Content of the interviews**
	• What concrete support would you like to have?<br>• What information do you need?<br>• How do you as a manager contribute to the satisfaction and health of your employees?<br>• Do you know of "best practice" examples in which managers take on this role particularly effectively?<br>• Have you had any particularly positive or negative experiences?
**6. Interview questions in relation to work organization, and work process**	Key question<br>• Which problems in work organization and work processes are particularly critical and urgent for you?<br>Follow-up questions<br>• How are incoming orders organized in your company?<br>• Can you give an example of a successful process?
**7. Final question and conclusion**	• Are there any aspects that have not been mentioned but that you would like to address regarding job satisfaction and perceived stress?<br>• Thank you for participating in the interview

Table S2: Template for the documentation of the single interviews

Note: Translated to English by the authors for the purpose of publication. Original version was in German only.

Interviewer	
Date of the interview (DD.MM.YYYY)	
Length of interview (in minutes)	
Code of the interviewed person	
Type of interview (face-to-face, telephone interview)	
**Main topics of the interview**	**Summary of the interview notes**
Job satisfaction	
Teamwork and team relations	
Leadership	
Work organization /work process	
Transfer	
Other statements /observations	

Table S3: Template for the documentation of the focus group discussion

Note: Translated to English by the authors for the purpose of publication. Original version was in German only.

**Structural data**	
Number of persons (researchers)	
Number of focus group participants	
Date of the focus group	
Start of the focus group discussion (time)	
End of the focus group discussion (time)	
**Description of each participant**	
Work activity	
Role in the institution	
Link to MSEs/SMEs	
Other work experience	
Work activity	
**Main topics**	**Summary of discussion round 1**
Leadership, selfcare, and team care	
Team relations and communication	
Work organization and work process	
Other statements /questions	
Observations	
**Main topics**	**Summary of discussion round 2**
Transfer	
Other statements /questions	
Observations	

**Main topics**	**Summary - Synthesis**
Summary of the two discussion rounds	
Summary and outlook	
Flash round	

Table S4: Schedule of the focus group discussion

Duration	Content
20 minutes	**Welcome and introductions**<br>• Project partners<br>• Experts for the focus group discussion
20 minutes	**Introduction of the IMPROVE*job* study**<br>• Aim and research questions of the study<br>• Methodological approach within the four sub studies of the IMPROVE*job* study<br>• Framework of the IMPROVE*job* study
45 minutes	**First discussion round**<br>• General input impulse<br>• First discussion round about general leadership challenges in MSE/SME settings
10 minutes	**Break**
45 minutes	**Second discussion round**<br>• General input impulse<br>• Second discussion round about possible transfer options in other MSE/SME settings
40 minutes	**Synthesis of the overall results and final evaluation with the online questionnaire**<br>• Summary<br>• Short feedback round with final statements of the experts<br>• Thanks for the participation<br>• Possibility to fill out the online questionnaire

Table S5: Items of the online questionnaire

Note: Translated to English by the authors for the purpose of publication. Original version was in German only.

Items	Possible answer options
1. The workshop was well organized.	1=Disagree strongly<br>2=Disagree<br>3= Partly/partly<br>4=Agree<br>5=Agree strongly
2. The workshop has promoted a mutual exchange between the participants.	1=Disagree strongly<br>2=Disagree<br>3= Partly/partly<br>4=Agree<br>5=Agree strongly
3. I am satisfied with the selection of topics during the workshop.	1=Disagree strongly<br>2=Disagree<br>3= Partly/partly<br>4=Agree<br>5=Agree strongly
4. In your opinion, were the impulses helpful in initiating discussion regarding the topics	1=Yes<br>2=No<br>3= Don't know
5. Can you pick up anything from the workshop for your day-to-day work?	1=Yes<br>2=No<br>3= Don't know
6. Overall, how satisfied are you with the workshop?	1=Very dissatisfied<br>2=Dissatisfied<br>3= Partly/partly<br>4=Satisfied<br>5=Very satisfied
